# Supplementary material for: Pre-existing visual responses in a projection-defined dopamine population explain individual learning trajectories
Source: Curr Biol. Author manuscript; Available in PMC 2024 Nov 27. (PMC11579926; doi:10.1016/j.cub.2024.09.045)
Supplement: Figure S1-S5 and Table S1-S2 [file EMS199720-supplement-Figure_S1_S5_and_Table_S1_S2.pdf]

Current Biology, Volume *34*

Supplemental Information

Pre-existing visual responses in a projection-defined dopamine population explain individual learning trajectories

Alejandro Pan-Vazquez, Yoel Sanchez Araujo, Brenna McMannon, Miranta Louka, Akhil Bandi, Laura Haetzel, Mayo Faulkner, International Brain Laboratory, Jonathan W. Pillow, Nathaniel D. Daw, and Ilana B. Witten

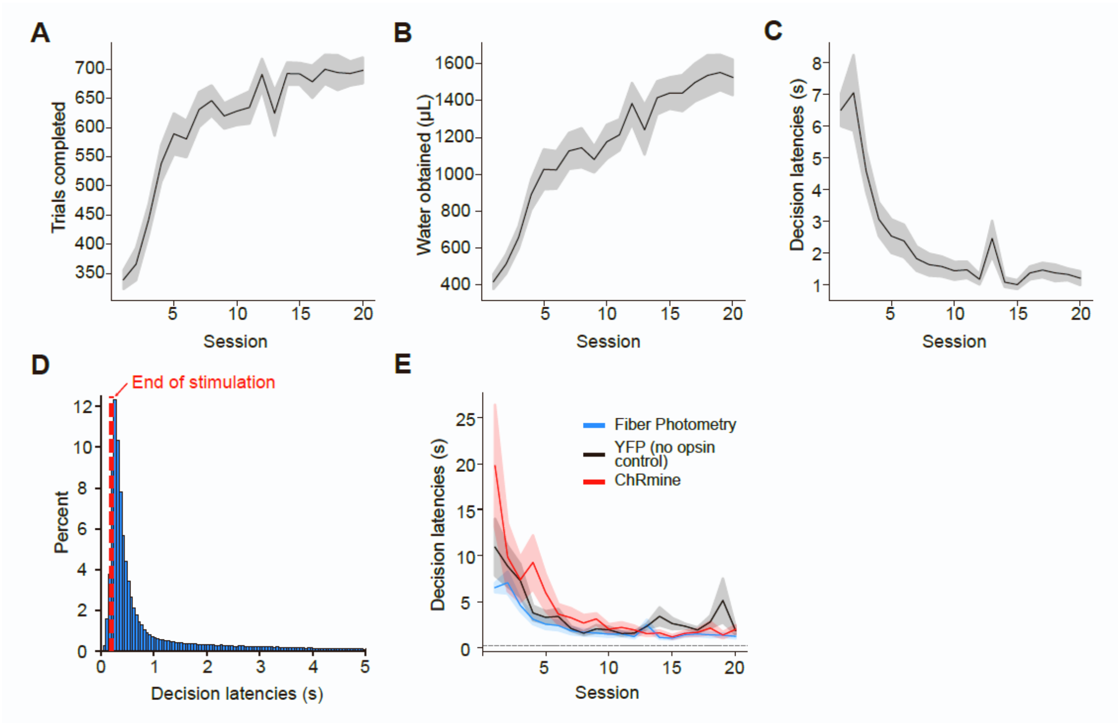

Figure S1 | Session statistics throughout training, including sessions with optogenetic stimulation (related to figure 1 and 4). (A) Trials completed, **(B)** Total water obtained across sessions. **(C)** Decision latencies (time between the go cue and the outcome delivery) across sessions. **(D)** Histogram of decision latencies across all mice in Figures 1-3 (*n=22*). Red line denotes the end of the optogenetic stimulation trains (0.2 s from the go cue) in Figure 4. **(E)** Comparison of decision latencies across sessions across ChRmine stimulation (red, *n=7*, Figure 4), no opsin control (YFP, black, *n=6*, Figure 4) and the fiber photometry (blue, *n=22*, Figure 1-3) cohorts. Dashed line represents the end of the optogenetic stimulation (200 ms). Across all panels, lines and shading represent mean +/- s.e.m across mice.

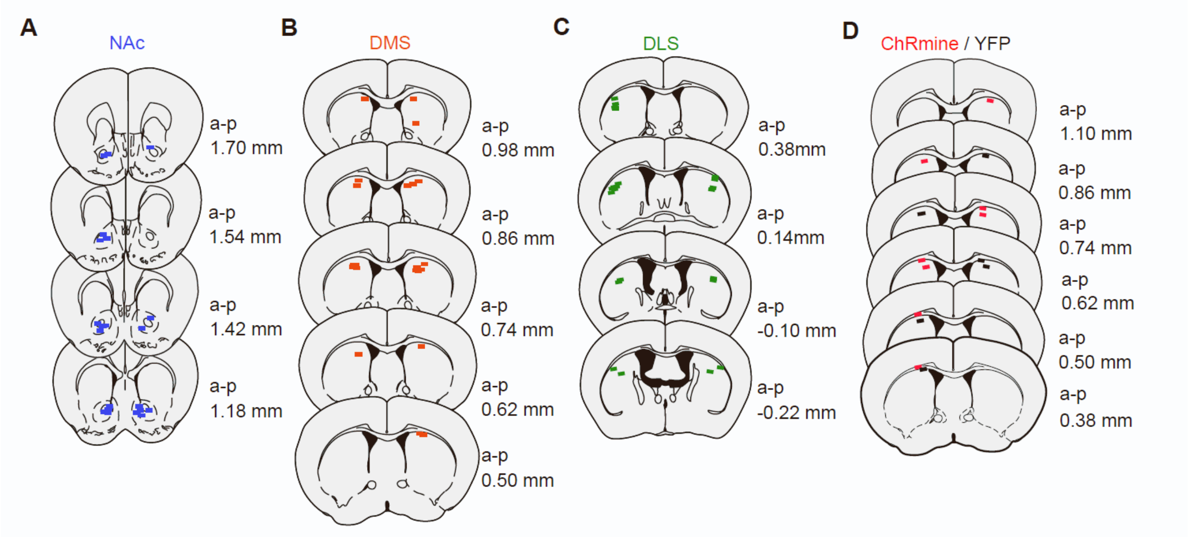

Figure S2 | Optical fiber location for dopaminergic terminal recordings and terminal optogenetic stimulation (related to Figure 2 - 4). Recovered fiber tip locations for the fiber photometry recordings in Figures 2-3 in **(A),** NAc, **(B),** DMS and **(C),** DLS. Each line (200μm) represents a fiber tip and their color relays their assigned striatal subregion: Blue - NAc, Orange -DMS, Green - DLS. **(D)** Recovered fiber tip locations for the optogenetic terminal stimulation experiment in Figure 4. Each line (300μm) represents a reconstructed fiber tip and their color relays their assigned group: Black - YFP (no opsin control) or Red - ChRmine. All fibers were located to the closest 100 μm section in the Paxinos-Franklin atlas [88]. Sections are ordered by anterior-posterior (a-p) distance from Bregma.

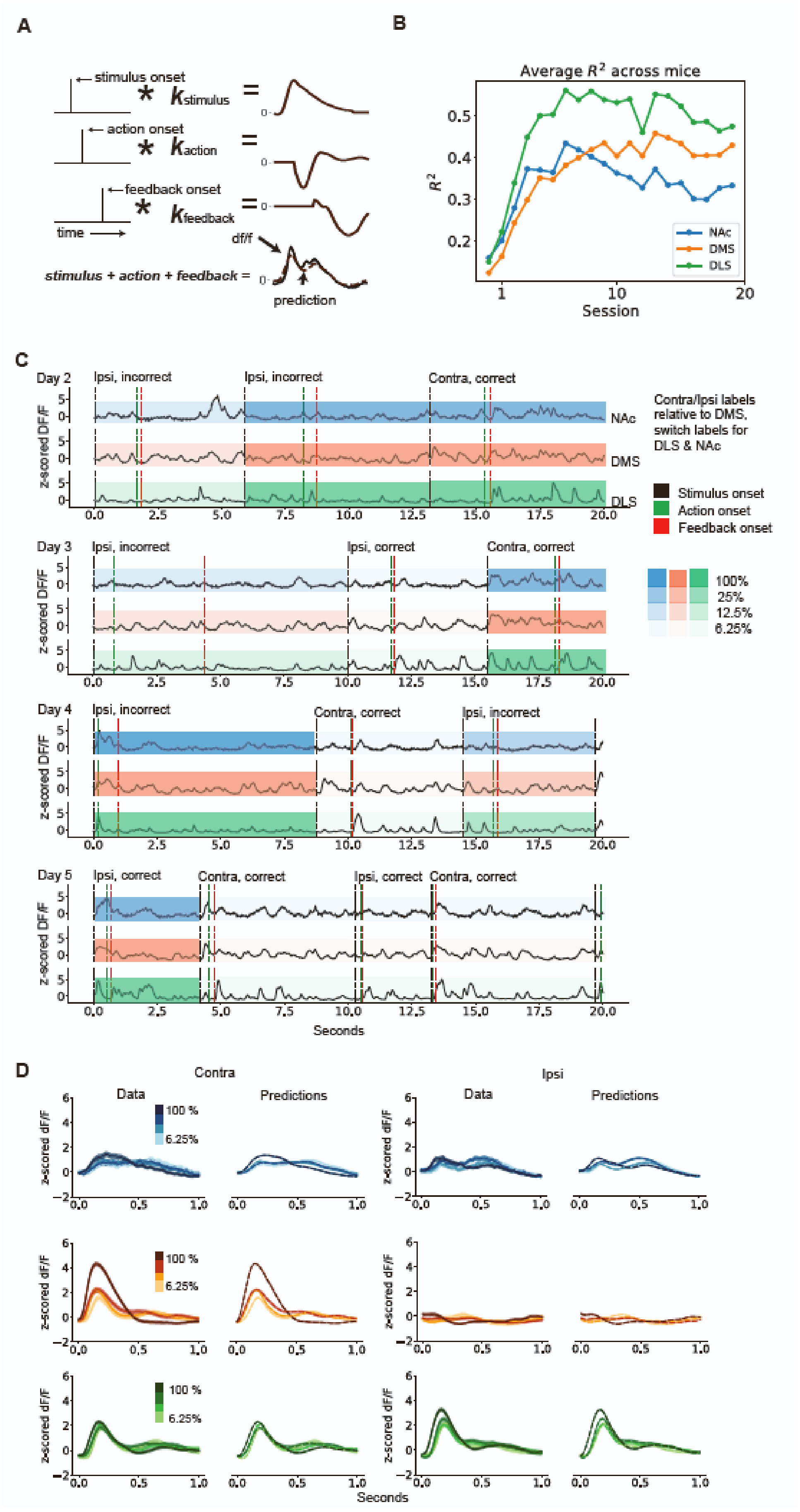
A
stimulus onset
$k_{stimulus}$
action onset
k_{action}
feedback onset
$k_{feedback}$
time
df/f
stimulus + action + feedback =
prediction
B
Average R^2 across mice
R^2
Session
NAc
DMS
DLS
C
Day 2
Ipsi, incorrect
Ipsi, incorrect
Contra, correct
z-scored DF/F
NAc
DMS
DLS
Contra/Ipsi labels relative to DMS, switch labels for DLS & NAc
Stimulus onset
Action onset
Feedback onset
100%
25%
12.5%
6.25%
Day 3
Ipsi, incorrect
Ipsi, correct
Contra, correct
Day 4
Ipsi, incorrect
Contra, correct
Ipsi, incorrect
Day 5
Ipsi, correct
Contra, correct
Ipsi, correct
Contra, correct
Seconds
D
Contra
Ipsi
Data
Predictions
z-scored dF/F
100 %
6.25%
Seconds

Figure S3 | Encoding model schematic & average explained variance (related to Figure 2)
(A) Encoding model schematic (see Methods for details). We convolved delta functions defining task relevant events such as stimulus onset, action onset, and feedback onset with temporal kernels of those events, then summed up all components to get the predicted response. Example 100% contrast trial is shown. **(B)** Explained variance in the fluorescence data (dF/F) by the model predictions, averaged across mice per training session. R^2 is the variance explained across all trials within a session (from stimulus onset to 1 second after feedback for each trial). **(C)** For an example animal, across 4 consecutive days, z-scored fluorescence traces on trial 200 for a contiguous span of 20 seconds. Dashed vertical lines denote event onsets (black) stimulus, (green) action, and (red) feedback. Shading strength indicates stimulus contrast strength, and shading color indicates region. Ipsi/Contra labels are written with respect to the DMS, thus for NAc and DLS, a Contra label indicates an Ipsi trial. **(D)** PSTH of the stimulus onsets for an example mouse / day across regions. Solid lines correspond to the z-scored fluorescence data, dashed lines are the PSTHs of the encoding model predictions.

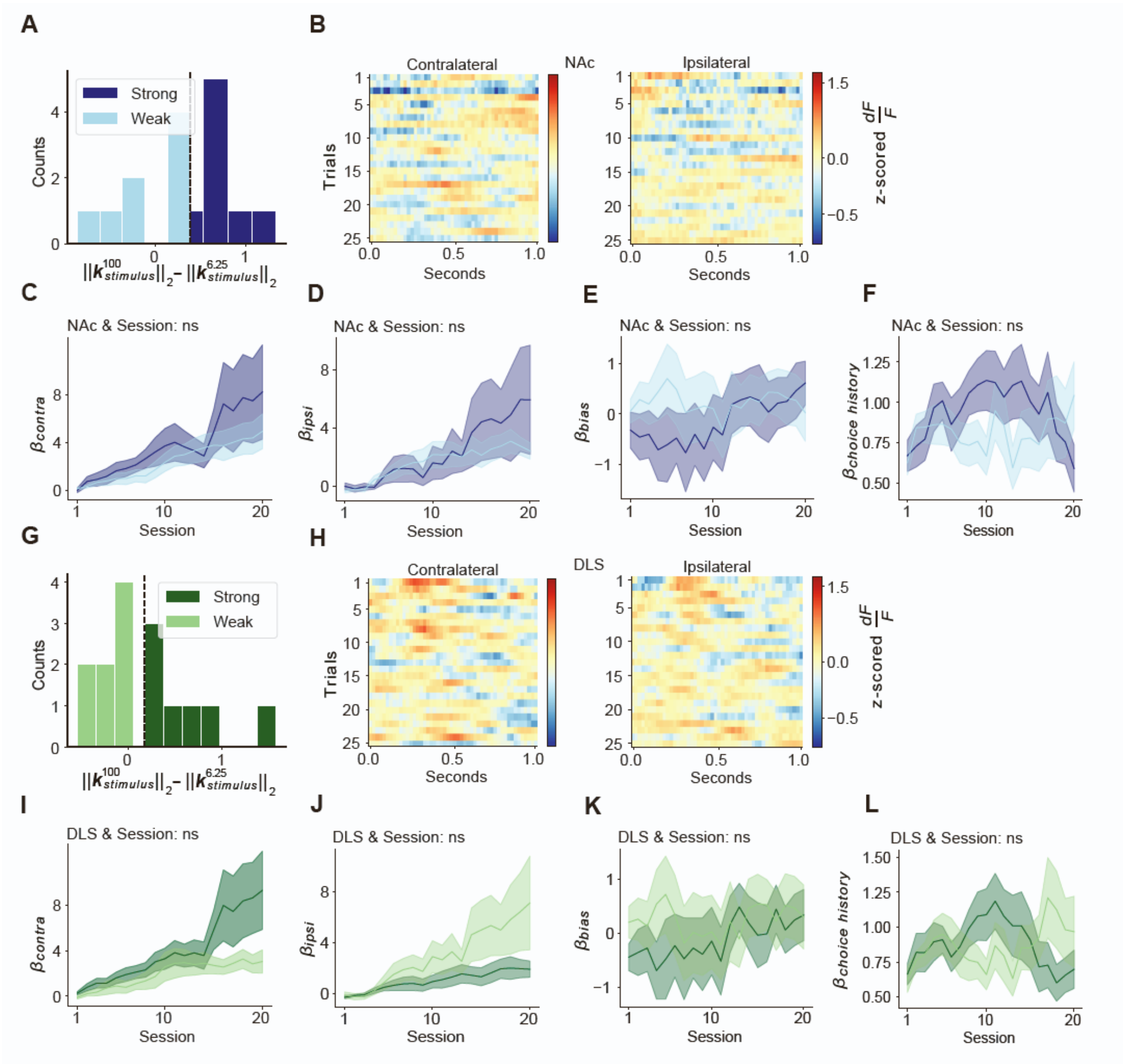

Figure S4 | Pre-existing visual responses in NAc & DLS DA (related to Figure 3).
(A) Histogram across mice of contrast-dependent NAc DA stimulus responses on session 0, quantified as the difference in the L2-norm of the highest and lowest contrast contralateral stimulus, colored by weak (light blue: < median) and strong (dark blue: > median). **(B)** Heatmap of NAc DA stimulus responses on Session 0 to 100% contrast stimuli for the first 25 trials, averaged across mice. **(C)** Contralateral stimulus sensitivity weights from the behavioral model, for mice with strong versus weak contrast-dependent stimulus NAc DA responses during session 0 (subdivision of mice shown in (a)). Lines and shading represent mean ± SEM. No significant interaction (ns) between NAc stimulus response on session 0 & session. **(D)** Same as (C), except for the ipsilateral stimulus weight from the behavioral model. No significant interaction (ns) between NAc stimulus response on session 0 and session. **(E)** Same as (C, D), but for the bias weights from the behavioral model (transformed such that positive means contralateral bias). No significant interaction (ns) between NAc stimulus response on session 0 & session. **(F)** Same as (C, D, E) but for the choice history weights from the behavioral model. No significant interaction (ns) between NAc stimulus response on session 0 & session. **(G)** Histogram across mice of contrast-dependent DLS DA stimulus responses on session 0, quantified as the difference in the L2-norm of the highest and lowest contrast contralateral stimulus, colored by weak (light green: < median) and strong (dark green: > median). **(H)**

Heatmap of DLS DA stimulus responses on Session 0 to 100% contrast stimuli for the first 25 trials, averaged across mice. **(I)** Contralateral stimulus sensitivity weights from the behavioral model, for mice with strong versus weak contrast-dependent stimulus DLS DA responses during session 0 (subdivision of mice shown in (A)). No significant interaction (ns) between DLS stimulus response on session 0 & session. Lines and shading represent mean ± SEM. **(J)** Same as (I), except for the ipsilateral stimulus weight from the behavioral model. No significant interaction (ns) between DLS stimulus response on session 0 &session. **(K)** Same as (I, J), but for the bias weights from the behavioral model (transformed such that positive means contralateral bias). No significant interaction (ns) between DLS stimulus response on session 0 & session. **(L)** Same as (I, J, K) but for the choice history weights from the behavioral model. No significant interaction (ns) between DLS stimulus response on session 0 & session. In all panels, *n*=18 mice.

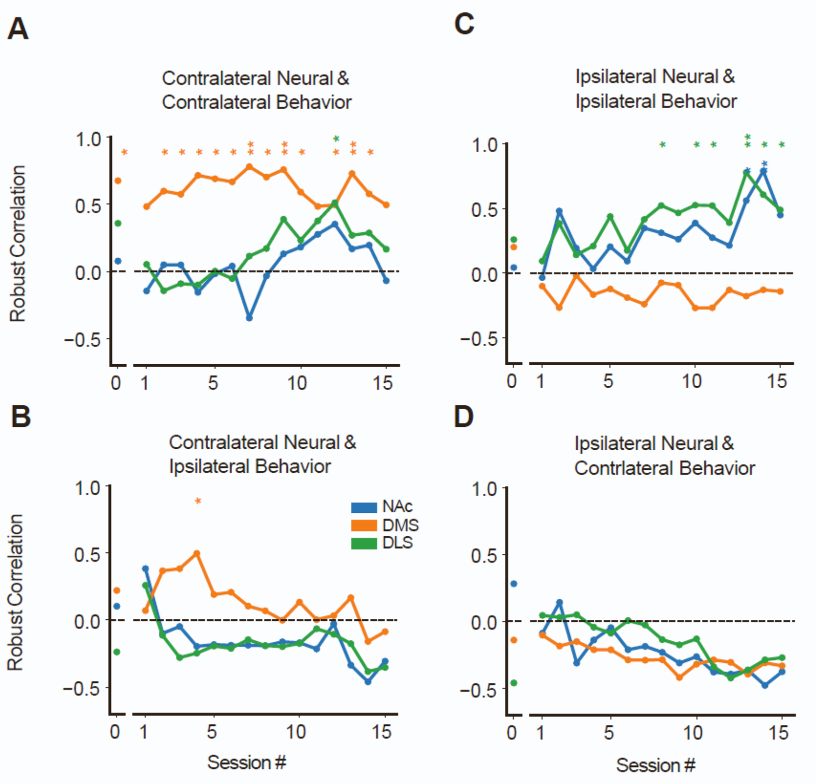

Figure S5 | Throughout training, contrast-dependent contralateral DMS DA stimulus responses predict contralateral stimulus-dependent performance during sessions 16-20 (related to Figure 3). (A) For each region, and for each session, correlation across animals of the average contralateral stimulus sensitivity weight from the behavioral model at the end of training (sessions 16-20) with the contrast-dependence of the contralateral stimulus kernel (difference of L2-norm of the highest and lowest contrast contralateral stimulus response). Session 0 denotes the pre-exposure session before the start of training, described in Figure 3. **(B)** Same as a, however for contralateral neural weights and ipsilateral behavioral weight estimates. **(C)** Same as (A, B), however using Ipsilateral for both neural and behavioral weight estimates. **(D)** Same as (A, B, C), however using ipsilateral neural weights and contralateral behavioral weights. In all panels, correlations and p-values are computed with robust regression, as described in the Statistical Analysis section of Methods.

	P-value	T-value	DF	SE	N
**Contra correlations: test against 0**					
**NAc**	0.0002	4.4	21	0.06	22
**DMS**	<1e-07	7.9	21	0.06	22
**DLS**	0.001	3.8	21	0.08	22
**Ipsi correlations: test against 0**					
**NAc**	0.0008	3.9	21	0.08	22
**DMS**	0.565	0.58	21	0.08	22
**DLS**	0.0013	3.69	21	0.07	22
**Contra & Ipsi comparison: test difference in means against 0**					
**NAc**	0.6523	-0.45	21	0.11	22
**DMS**	0.0002	4.4	21	0.1	22
**DLS**	0.7054	0.38	21	0.11	22
**Contra minus Ipsi: test difference of differences in means against 0**					
**NAc & DMS**	0.0104	2.8	21	0.18	22
**DMS & DLS**	0.0516	2.06	21	0.2	22

Table S1 | Statistics. Related to Figure 2F

Statistics for Figure 4C (individual coefficients)<br>$correct \sim 1 + session * cohort * contra + (1 + session * contra \mid mouse)$				
**Variable**	**Coefficient**	**Std. Error**	**z**	**Pr(>|z|)**
**Intercept**	0.598551	0.093143	6.43	<1e-09
**session: middle**	0.0968696	0.0682439	1.42	0.1558
**session: late**	0.0780368	0.0923531	0.84	0.3981
**cohort: chrmine**	-0.276154	0.126932	-2.18	0.0296
**contra: ipsi**	-0.19325	0.17514	-1.1	0.2699
**session: middle & cohort: chrmine**	0.0453335	0.0930008	0.49	0.6259
**session: late & cohort: chrmine**	0.195543	0.125856	1.55	0.1203
**session: middle & contra: ipsi**	0.0907977	0.139328	0.65	0.5146
**session: late & contra: ipsi**	0.227952	0.183541	1.24	0.2142
**cohort: chrmine & contra: ipsi**	0.51202	0.238676	2.15	0.0319
**session: middle & cohort: chrmine & contra: ipsi**	-0.229823	0.189872	-1.21	0.2261
**session: late & cohort: chrmine & contra: ipsi**	-0.432753	0.250124	-1.73	0.0836
**Variable coding:** See variable coding in statistical analysis section of Methods.				

Statistics for Figure 4C (ANOVA, type 3)<br>$correct \sim 1 + session * cohort * contra + (1 + session * contra \mid mouse)$				
**Variable**	**DOF**	**Res.DOF**	**F value**	**Pr(>|F|)**
**Intercept**	1	498	40.3425	<1e-09
**session**	2	10	0.9958	0.4033
**cohort**	1	10	4.624	0.057
**contra**	1	498	1.1894	0.276
**session & cohort**	2	498	1.2345	0.2919
**session & contra**	2	498	0.7554	0.4703
**cohort & contra**	1	498	4.4959	0.0345
**session & cohort & contra**	2	498	1.5	0.2241
**Variable coding:** See variable coding in statistical analysis section of Methods.				

Table S2 | Statistics. Related to Figure 4
